# BAP31, a newly defined cancer/testis antigen, regulates proliferation, migration, and invasion to promote cervical cancer progression

**DOI:** 10.1038/s41419-018-0824-2

**Published:** 2018-07-18

**Authors:** Erle Dang, Shuya Yang, Chaojun Song, Dongbo Jiang, Zichao Li, Wei Fan, Yuanjie Sun, Liang Tao, Jing Wang, Tingting Liu, Chunmei Zhang, Boquan Jin, Jian Wang, Kun Yang

**Affiliations:** 1 Department of Immunology, the Fourth Military Medical University, Xi’an, 710032 Shaanxi People’s Republic of China; 2 Department of Dermatology, Xijing Hospital, the Fourth Military Medical University, Xi’an, 710032 Shaanxi People’s Republic of China; 3 School of Life Science, Northwestern Polytechnic University, Xi’an, 710072 Shaanxi People’s Republic of China; 4 Department of Obstetrics and Gynecology, Xijing Hospital, the Fourth Military Medical University, Xi’an, 710032 Shaanxi People’s Republic of China

## Abstract

Malignant tumors typically undergo an atavistic regression characterized by the overexpression of embryonic genes and proto-oncogenes, including a variety of cancer/testis antigens (CTAs) that are testis-derived and are not expressed or expressed in trace amounts in somatic tissues. Based on this theory, we established a new method to identify unknown CTAs, the spermatogenic cells-specific monoclonal antibody-defined cancer/testis antigen (SADA) method. Using the SADA method, we identified BAP31 as a novel CTA and confirmed that BAP31 expression is associated with progression and metastasis of several cancers, particularly in cervical cancer. We found that BAP31 was significantly upregulated in stage I, II, and III cervical cancer patients and highly correlated with poor clinic outcomes. We further demonstrated that BAP31 regulates cervical cancer cell proliferation by arresting the cell cycle at the G0/G1 stage and that depletion of BAP31 inhibits hyper-proliferation. Moreover, depletion of BAP31 inhibits cervical cancer cell invasion and migration by regulating the expression and subcellular localization of Drebrin, M-RIP, SPECC1L, and Nexilin, and then affect the cytoskeleton assemblage. Finally, the depletion of BAP31 prevents cervical cancer progression and metastasis in vivo. These findings provide a new method for identifying novel CTAs as well as mechanistic insights into how BAP31 regulates cervical cancer hyper-proliferation and metastasis.

## Introduction

The immune system can recognize tumor antigens in cancer patients, and that therapeutic manipulation of immunity can control tumor growth^1^. In the early 1990s, Boon and colleagues successfully cloned the first tumor antigen, MAGEA1, using T-cell-based approach^2^, and MAGEA1 could have elicited a spontaneous cytotoxic T lymphocyte (CTL) response in the autologous melanoma patients^3^. Subsequently, a range of different human antigens, including proteins derived from tumor-specific mutant genes, alternatively initiated proteins or normal proteins, which display aberrant quantitative or qualitative expression in tumor cells, have been identified^4^. In addition, some of the tumor antigens have been used as diagnostic and prognostic markers in several types of cancers^5^. However, the weak antigenicity of these tumor antigens and a lack of reliable methodologies have restricted their development in cancer therapy.

Cancer/testis antigens (CTAs), a group of testis-derived proteins, are normally expressed only in the male testis and are dramatically increased in various types of cancer tissues^6^. Because of their restricted expression in immune-advantaged organs, CTAs are attractive targets for anticancer immunotherapy, and some CTAs are currently being used as biomarker for the diagnosis and prognosis of cancer or as targets in clinical trials for vaccine immunotherapy^7^. However, the expression of CTAs such as the MAGE family and NY-ESO-1 are limited to those patients with a particular tumor type, which restrict their development as an effective supplement to conventional cancer treatments^8,9^. Hence, extensive effort is required to develop more effective strategies for identifying highly immunogenic and cancer-specific tumor antigens for future treatments. In the present study, we report a new method to screen potential CTAs, the spermatogenic cells-specific monoclonal antibody-defined cancer/testis antigens (SADA) method, and succeed in discovering five new molecules with CTA expression patterns. Subsequently, we defined one of the candidate CTA is B-cell receptor-associated protein 31 (BAP31).

BAP31 is a 28-kDa integral membrane protein in the endoplasmic reticulum^10,11^. BAP31 consists of an N-terminal transmembrane domain and a C-terminal cytoplasmic region, which forms a coiled-coil^12^. BAP31 functions as an escorting factor in the sorting of integral endoplasmic reticulum (ER) membrane proteins, including major histocompatibility class I molecules^13^, immunoglobulin D^11^, cystic fibrosis transmembrane regulator^14^, cellubrevin^10^, cytochrome P450^15^, and CD11b/CD18^16^. Furthermore, BAP31 can also mediate the degradation and retrotranslocation of mutant CRTF protein by binding to the Sec61 preprotein translocon, suggesting that BAP31 controls the fates of its bound clients for ER export/retention/degradation^14^. In addition to its role in ER protein trafficking, BAP31 has been reported to be involved in a number of apoptotic pathways after the cleavage of its C-terminus by caspase-8 and functions as a regulator of apoptosis through an interaction with Bcl-2 or Bcl-XL and caspase-8^17–19^. Considering all these important capabilities of BAP31, recent studies have uncovered the critical factors responsible for the survival and stemness of human embryonic stem cells and the proliferation of human papillomavirus (HPV)-positive keratinocytes^20,21^, which suggested that BAP31 might be involved in the pathogenesis of HPV-related cancers.

In this study, we confirmed that BAP31 serves as a novel CTA and that its expression as correlated with cervical tumor progression and patient prognosis. In addition, we demonstrated that BAP31 aids the proliferation and metastasis of cervical cancer cells by arresting the cell cycle and regulating the expression and subcellular localization of metastasis-related proteins. Finally, we proved that suppression of BAP31 could prevent cervical cancer progression and metastasis in vivo.

## Results

### Identification of BAP31 as a new CTA using SADA method

**Fig. 1:**
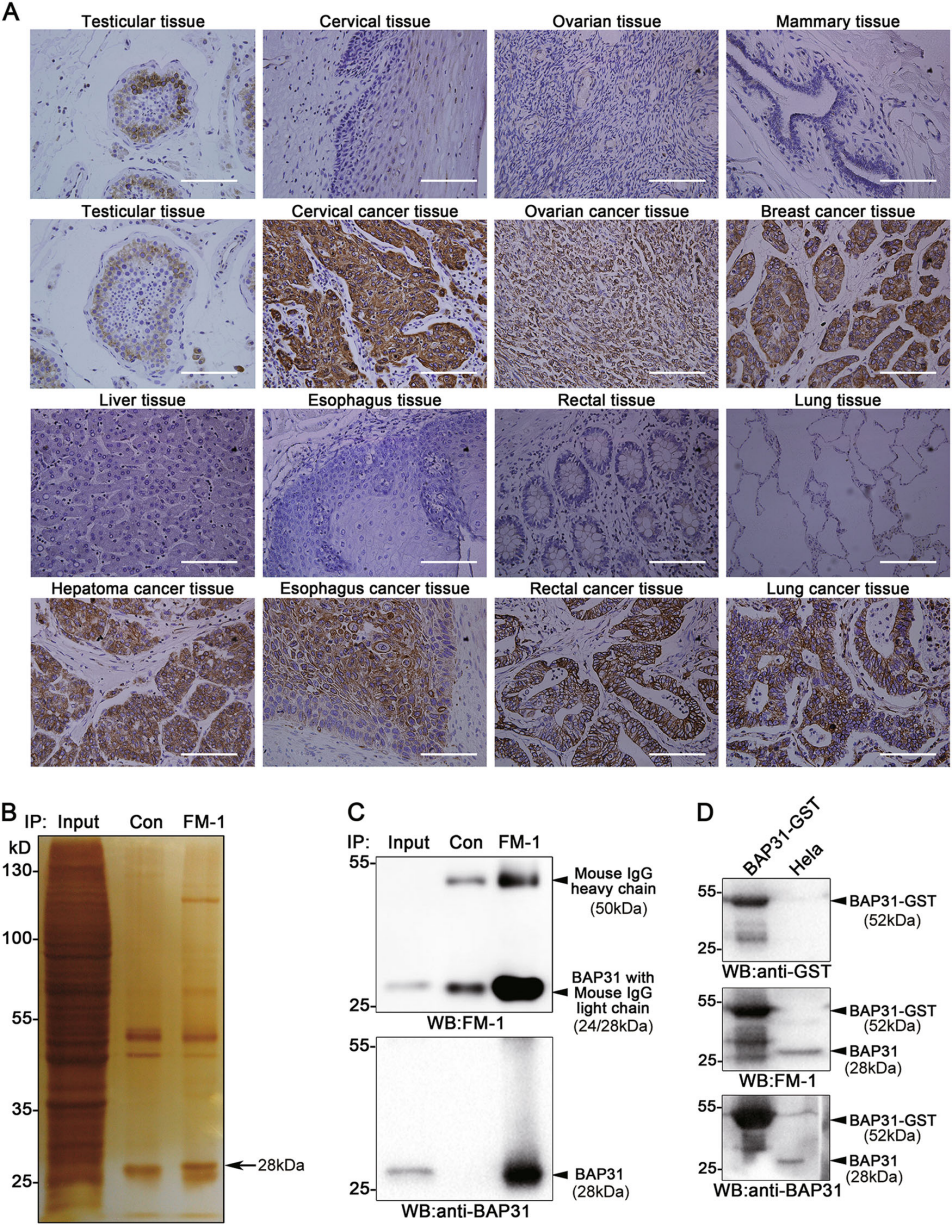
Identification of BAP31 as a new CTA using the spermatogenic cell-specific monoclonal antibody-defined cancer/testis antigen (SADA) method. The SADA method was employed to generate candidate antibodies against cancer/testis antigens. Spermatogenic cells purified from donated testis tissues were used to immunize BALB/c mice, and hybridomics procedures were used to generate candidate antibodies against Spermatogenic cells. **a** The antigen recognized by FM-1 was confirmed as a CTA by immunohistochemical staining of testicular tissues, somatic normal tissues, and related cancer tissues. The scale bars represent 100 μm. **b** SDS-PAGE electrophoresis and silver staining of the FM-1 immunoprecipitates from HeLa cells lysates revealed that an ~ 28 kDa protein band (shown by arrow) was enriched in the gel. **c** Immunoprecipitates from HeLa cells lysates treated with FM-1 were analyzed with an FM-1 antibody and anti-BAP31 antibody by western blots. **d** CHO cells were transfected with the BAP31-GST plasmid pcDNA3.1. Cell lysates were subjected to western blotting with FM-1, anti-GST, and anti-BAP31 antibodies, respectively. HeLa cells lysates were loaded as a positive control

CTAs expression is limited to male germ cells in healthy adults, but ectopic expression has been observed in tumor cells from multiple types of human cancers^7^. Based on these theory, we developed the SADA method as a new method to identify unknown CTAs (Supplementary Fig S1). BALB/c mice were immunized with human spermatocytes/spermatogonia purified from donated testis tissue with a purity > 80%, as determined by immunohistochemical staining with LEAG-1 antibody (Supplementary Fig S2A and B). The mAbs against spermatogenic cells were then raised using hybridomics. Five candidate antibodies were generated with expression patterns similar to CTAs, as determined by immunohistochemical staining of a tissue array including testis, healthy and tumor tissues (Supplementary Table S1). Strikingly, antibody FM-1 detected highest positive rate than other four antibodies in testicular tissues or several types of tumor tissues, including cervical, ovarian, breast, liver, esophageal, rectal and, lung cancers, and no or weak expression in paired normal tissues (Fig. 1a). Hence, we further investigated the target of FM-1 and its function in cancer.

To identify the antigen recognized by FM-1, positively stained HeLa cells were cultured and lysed for immunoprecipitation with FM-1 (Supplementary Fig S3). An ~ 28-kDa protein band was enriched in both the polyacrylamide gel electrophoresis (PAGE) gel and western blots (Fig. 1b, c up). After mass spectrometry analysis and protein database searching, we identified the target protein as BAP31 (Supplementary Fig S2C), which was confirmed by western blotting using an anti-BAP31 antibody (Fig. 1c down). To confirm that BAP31 is specifically recognized by FM-1, the human BAP31 expression plasmid BAP31-GST was constructed and transfected into Chinese hamster ovary (CHO) cells, and then the cell lysates were subjected to western blotting to detect the expression of BAP31-GST protein (Fig. 1d). The same lysates were subjected to western blotting with anti-GST, FM-1, or anti-BAP31 antibodies; HeLa cell lysates were used as a positive control. The BAP31-GST proteins was detected in CHO cells transfected with the BAP31-GST plasmid but not in untransfected cells. Thus, BAP31 was identified as a new CTA using the SADA method.

### BAP31 expression is increased in cervical cancer patients and positively correlated with survival

To further validate BAP31 as a cancer antigen, we first searched the Oncomine database for the clinical relevance of BAP31 in cancers. We observed that BAP31 levels were significantly increased in tumor tissues from patients with cervical squamous cell carcinoma compared with the normal controls (Table 1).Similar increases in BAP31 were observed in other cancers, such as breast cancer, colorectal cancer melanoma, and lymphoma (data not shown). Meanwhile, western blot revealed that BAP31 was highly expressed in several cervical cancer cell lines (including Hela, Siha, Caski, and C33A) (Supplementary Fig S4). These data are consistent with our identification of BAP31 as a CTA in various human cancers.

**Fig. 2:**
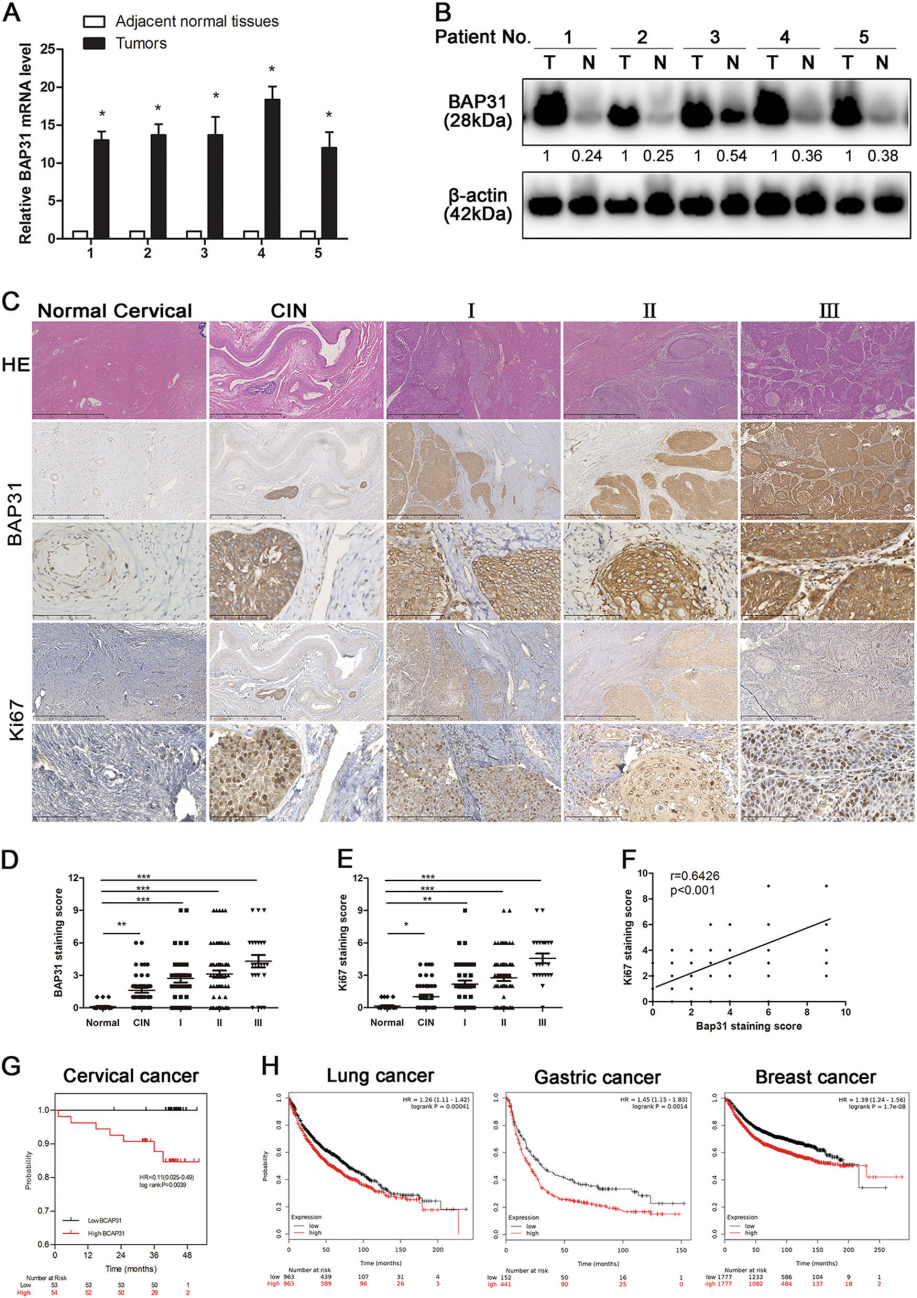
BAP31 expression is increased in cervical cancer patients and positively correlated with survival. **a** BAP31 mRNA levels from paired cervical tumors and adjacent normal tissues were analyzed by real-time PCR. *p < 0.05 was considered significant for the cervical tumor vs adjacent normal tissues. **b** The expression of BAP31 in five paired cervical tumors and adjacent normal tissues was determined by western blotting. **c** Paraffin sections of normal cervical and cervical cancer tissues were subjected to H&E staining and immunohistochemical staining using BAP31 antibody. The scale bars represent 1 mm and 100 μm. **d, e** Immunohistochemistry analysis of BAP31 staining score in 26 normal cervical tissue and 161 cervical cancer tissues. *p* value was calculated by one-way ANOVA. *p < 0.05, **p < 0.01, and ***p < 0.001. **f** Correlation between tissue BAP31 level and Ki-67 staining score was tested by Spearman’s rank correlation analysis, with *r* and *p* values indicated. **g** Kaplan–Meier survival curve showing overall survival of cervical cancer patients with low (*n* = 53) or high (*n* = 54) levels of BAP31 expression. Patients’ survival data were derived from 107 cervical cancer patients, and 54 patients were loss to follow-up. **h** Kaplan–Meier survival curves of breast cancer, gastric cancer, and lung cancer with low and high BAP31 expression from the Kaplan–Meier Plotter database. (*p* < 0.01 by log-rank test)

To assess the clinical relevance of BAP31 in cervical cancer progression, we surgically collected paired cervical carcinoma samples and adjacent normal tissues from five patients to measure BAP31 expression. We found that, compared with the paired normal tissues, all five cervical carcinoma specimens had substantially increased BAP31 expression in the level of mRNA and protein (Fig. 2a, b). This increase was validated independently by immunohistochemical staining of BAP31 in paraffin sections from 161 cervical cancer cases (Fig. 2c, Table 2). We found that BAP31 was highly expressed in 135 (83.6%) cancer samples, with obvious expression at the invasive front, whereas no or weak expression was observed in normal or nonmalignant lesions of the cervix (Fig. 2d). High BAP31 expression correlated significantly with lymph node metastasis and more advanced histological grade. As Ki-67 is an excellent marker to determine cervical cancer diagnosis and prognosis, we also analyzed the correlation between BAP31 and Ki-67 expression in cervical cancer. In line with previous studies, our immunohistochemistry assay showed that Ki-67 expression was significantly increased in cervical cancer and correlated with clinical stage. More importantly, BAP31 expression was in positively association with Ki-67 expression in cervical cancer tissues (Fig. 2c, e, f).

To further investigated whether BAP31 could be a potential prognosticator for cervical cancer, we performed the survival analysis of the 107 patients (54 case were missed in the telephone follow-up). The patients were divided into two group as low and high group by the immunohistochemical staining scores, the Kaplan–Meier analysis showed that lower levels of BAP31 expression were associated with improved overall survival in patients with cervical cancers (Fig. 2g). Surprisingly, high levels of BAP31 expression were also closely associated with poor overall survival rates in patients with breast cancer, gastric cancer and lung cancer (Fig. 2h) in the Kaplan–Meier plotter database. All together, these results indicated that BAP31 might be an independent prognostic factor for cancer survival and suggested that the increased expression of BAP31 might play an important role in the progression of cervical carcinoma.

### Functional implication of BAP31 in the pathogenesis of cervical cancer

**Fig. 3:**
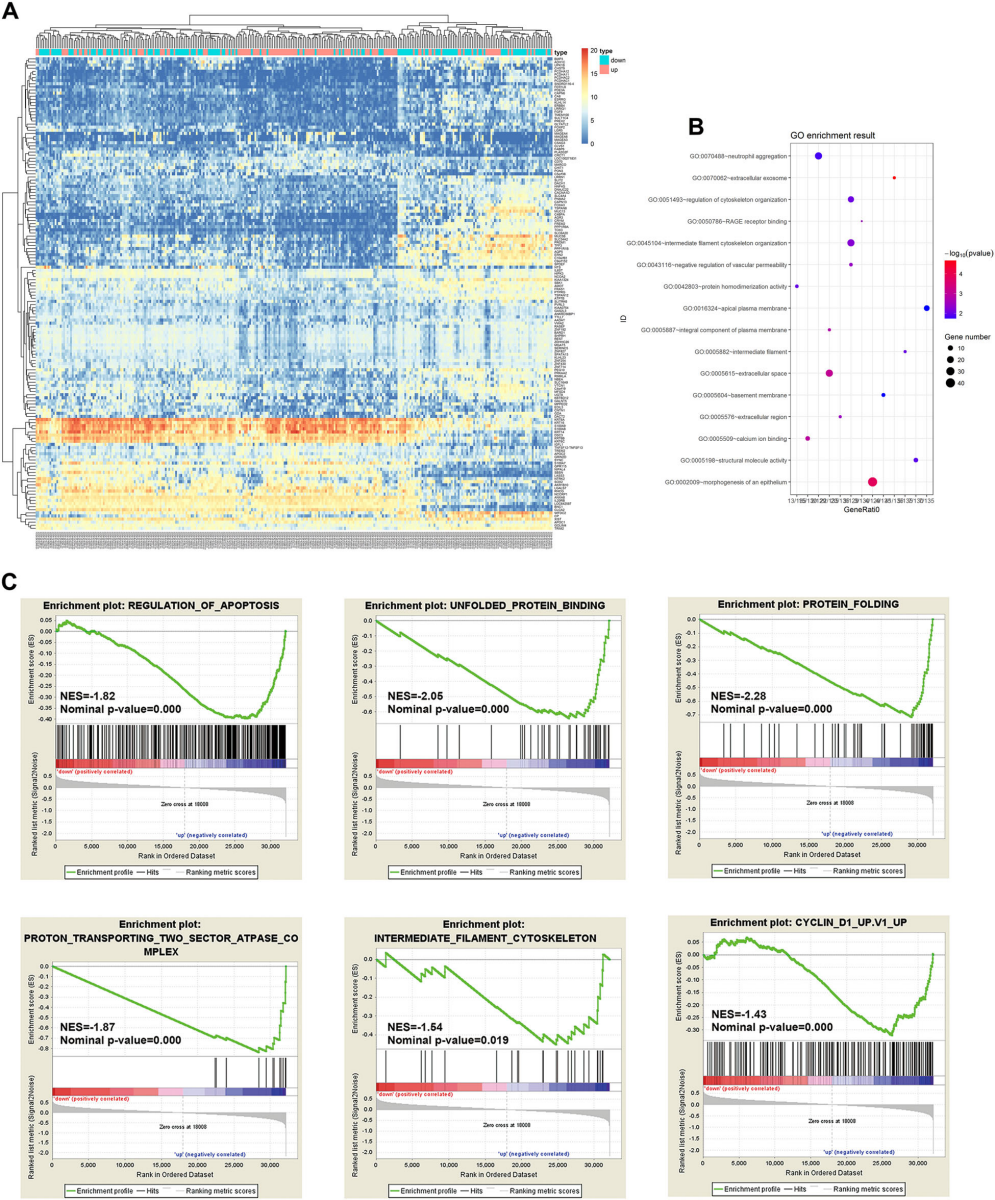
TCGA replication analysis. Genome-wide data were downloaded from TCGA for 303 available cervical cancer tumors. All the tumors were divided in two high and low BAP31 group by the BAP31 expression. **a** Heatmap representing genes that are significantly enriched for interaction with BAP31 (*p* < 0.01, Fisher’s exact test with Benjamini-Hochberg correction, fold-enrichment > 2). **b** GO enrichment analysis of mRNAs deregulated in high BAP31 group compared with low BAP31 group. **c** Gene Set Enrichment Analysis (GSEA) using stratified BAP31 transcript levels for genes upregulated in regulation of apoptosis, unfolded protein binding, protein folding, protein transporting, intermediate filament cytoskeleton, and cyclin D1

To explore the potential function of BAP31 in the pathogenesis of cervical cancer, we downloaded RNA-Seq expression profiles of 303 cervical cancer patients from TCGA database for bioinformatics analysis. Patients in each data set were classified into two groups of high and low BAP31 mRNA levels according to the median. After the initial analysis of all genes, a total of 152 genes were considered to be differentially expressed between high BAP31 group and low BAP31 group (cutoff of absolute log_2_FC > 1 and adjusted *p* value < 0.01) (Fig. 3a). Then we conducted functional enrichment analysis for upregulated and downregulated mRNA, respectively. The Gene ontology (GO) and Kyoto Encyclopedia of Genes and Genomes (KEGG) enrichment showed that all the 109 upregulated mRNA are mainly involved in many cellular function, such as cytoskeleton organization, extracellular space, and Neutrophil aggregation (Fig. 3b). To confirm the relationship between stratified BAP31 transcript levels and functional related genes, we performed Gene Set Enrichment Analysis (GSEA) on expression data from TCGA database. Stratified BAP31 transcript level were remarkably associated with the upregulated genes related to regulation of apoptosis, unfolded protein binding, protein folding, protein transporting, intermediate filament cytoskeleton, and cyclin D1, which consist with the GO and KEGG enrichment (Fig. 3c).

### Depletion of BAP31 inhibits the proliferation and invasion of cervical cancer cells in vitro

**Fig. 4:**
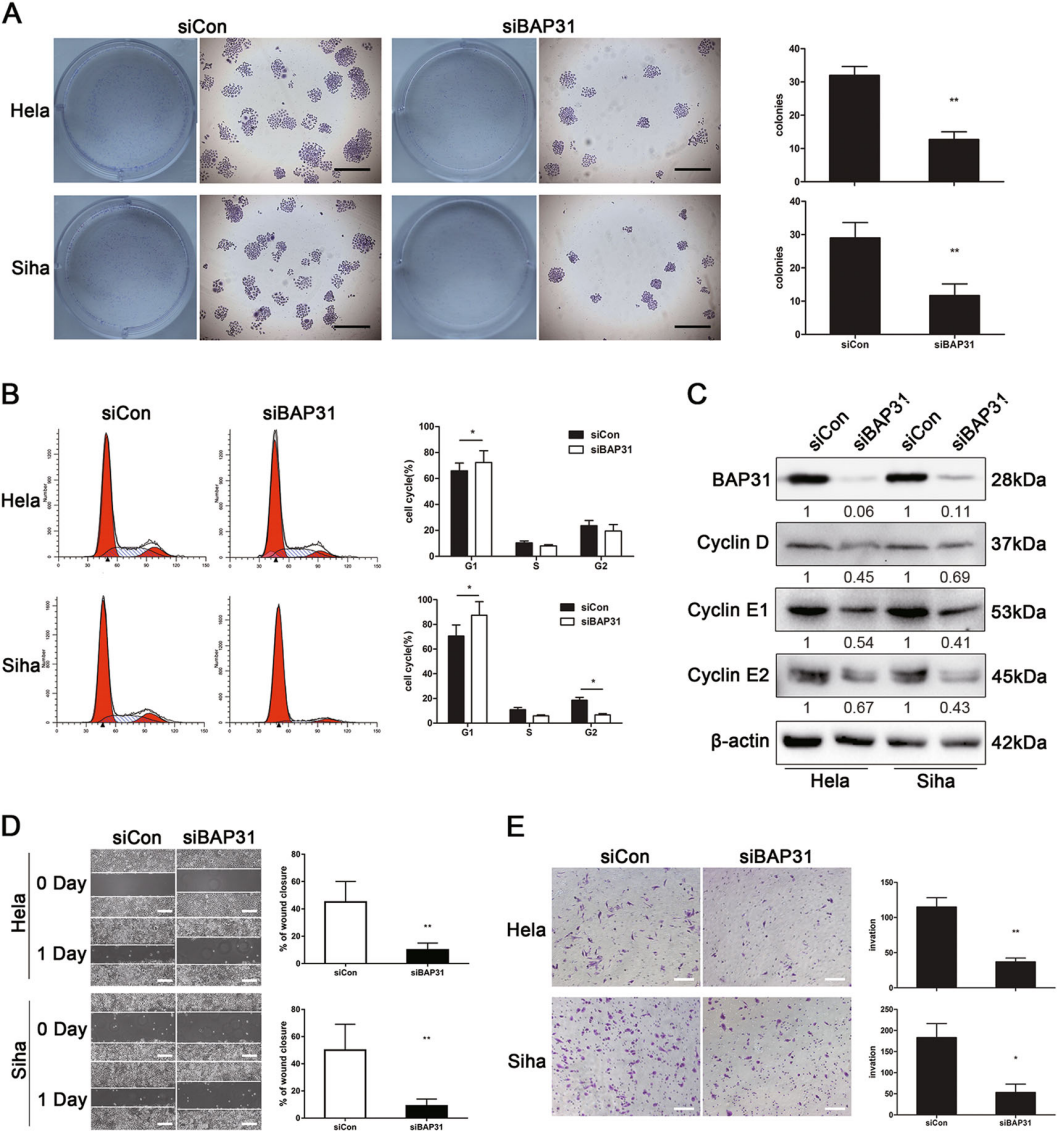
BAP31 depletion inhibits cervical cancer cells proliferation and invasion in vitro. **a** The proliferation of HeLa and SiHa cell lines was analyzed using a colony-formation assay after transfection with BAP31 or control siRNA. The results represented the means ± SD of triplicate independent experiments. The scale bars represent 100 μm. **b** Flow cytometric analysis of the HeLa and SiHa cell cycle distributions with PI at 48 h post transfection with BAP31 or control siRNA. The results are shown as the percentages of cells in G1, S, and G2 phases. *p < 0.05 was considered to be significantly different for the siBAP31 vs siCon group. **c** Western blotting results for the expression levels of BAP31, Cyclin D1, Cyclin E1, and Cyclin E2 in the control and the BAP31-depleted group of HeLa and SiHa cell lines. **d** Wound-healing assays were performed to evaluate the migration of HeLa and SiHa cells transfected with BAP31 or control siRNA. The scale bars represent 100 μm. **e** Transwell invasion assay of HeLa and SiHa cells treated as in (**a**). The results represent the means ± SD from five microscopic fields. The scale bars represent 100 μm. *p < 0.05 and **p < 0.01 were considered significantly different for the siBAP31 vs siCon groups

To further assess the effects of BAP31 on cancer growth, we went on to investigate whether BAP31 could influence the biologic behavior of cervical cancer cell lines. HeLa and SiHa cells were transfected with either BAP31 or control siRNAs, and BAP31 siRNA decreased BAP31 expression in the two cell lines by ~ 90% after transfection (Fig. 4c). First, colony-forming assays were performed to assess the proliferation of HeLa and SiHa cells after depletion of BAP31. The colony-formation efficiencies of BAP31-depleted HeLa and SiHa cells decreased by ~ 65 and 60%, respectively (*p* < 0.01 vs control siRNA) (Fig. 4a), suggesting that BAP31 expression is required for HeLa and SiHa cell proliferation. To examine the underlying mechanism, we measured the cell cycle distribution by propidium iodide incorporation and cell cycle-related protein expressions by western blotting. The proportions of cells in G0/G1 phase were increased in BAP31-depleted HeLa and SiHa cells (*p* < 0.05), whereas the proportions in S and G2/M phases decreased (*p* < 0.05) (Fig. 4b). In addition, the expression of cell cycle-related proteins, such as cyclin D, cyclin E1, and cyclin E2, were all significantly decreased in BAP31-depleted HeLa and SiHa cells. Therefore, the depletion of BAP31 inhibited the proliferation of cervical cancer cell lines by arresting the cell cycle at G0/G1 phase.

Considering high levels of BAP31 expression were correlated with clinical cervical tumor metastasis, we performed wound-healing assays and transwell invasion assays to determine if the depletion of BAP31 inhibits the cancer cell migration and invasion. The wound-healing assay showed that depletion of BAP31 delayed wound closure in HeLa and SiHa cells (Fig. 4d). Subsequent transwell assay showed that depletion of BAP31 inhibited the migration and invasion of compared with the control (Fig. 4e), suggesting that high levels of BAP31 expression promoted metastasis of cervical tumor cells. Taken together, these data imply that BAP31 may be a key promoter of cervical cell migration and invasiveness and thus accelerate the cancer progression.

### Depletion of BAP31 suppresses cervical cancer progression and metastasis in vivo

**Fig. 5:**
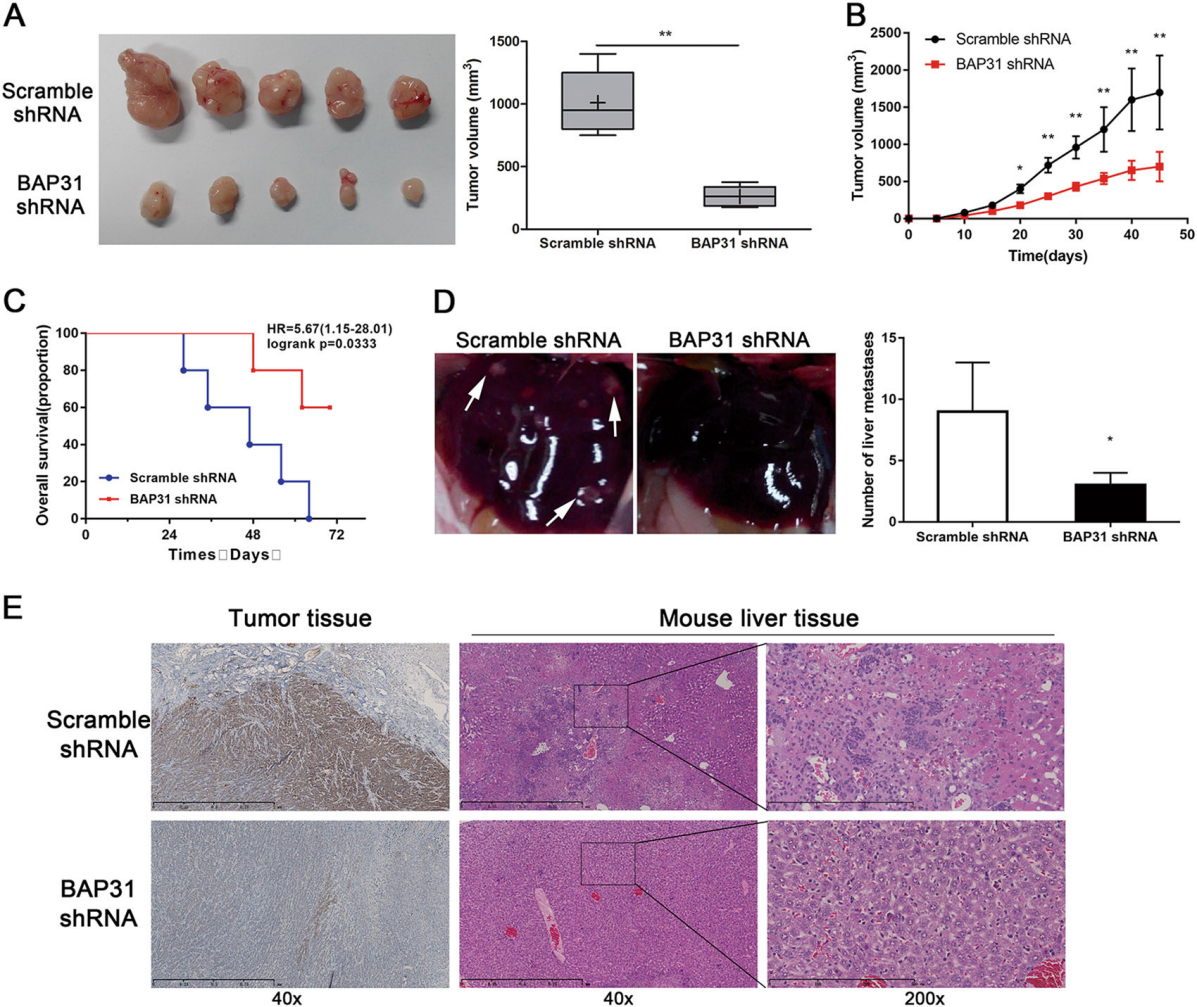
Depletion of BAP31 suppresses cervical cancer progression and metastasis in vivo. **a** Image of tumors and tumor volumes measured after 30 days. HeLa cells were stably transfected with BAP31 shRNA and injected subcutaneously into the left and right flanks of nude mice. HeLa cells transfected with control shRNA were used as controls and five mice were included in each group. The results represent the mean ± SD and **P < 0.05 was considered to be significantly different using Student’s *t* test. **b** Growth curve of xenografts of Hela cells transfected with BAP31 shRNA or Scramble shRNA. (*n* = 5, Mean ± SD) **c** Kaplan–Meier means curve representing the overall survival of the injected mice with BAP31-depleted or control HeLa cells. (*p* < 0.01 by log-rank test). **d** Representative pictures and statistics analysis of the tumor metastasis in livers. Arrows indicate tumor metastasis site. The results represent the mean ± SD and *P < 0.05 was considered to be significantly different using Student’s *t* test. **e** Immunohistochemical staining of BAP31 in tumor tissue from xenograft mouse model and detection of liver metastasis by HE staining. The scale bars represent 1 mm and 300 μm

Given that BAP31 upregulation was associated with advanced stages and worse clinical outcome of cervical cancer patients, we generated stable BAP31-depleted HeLa cells with shRNA vectors and injected them subcutaneously into the flanks of nude mice to construct a xenograft mouse model (HeLa cells transfected with scramble shRNA were used as a control). The BAP31-depleted group mice developed smaller tumors compared with the control group (Fig. 5a and b). In addition, depleted expression of BAP31 correlated with significantly increased survival of the xenografted mice (Fig. 5c), suggesting that depletion of BAP31 expression inhibited cancer progression in vivo. Furthermore, we observed metastasis of stable BAP31-depleted HeLa cells in the xenograft mouse model. Consistent with the findings in vitro, liver metastasis was significantly inhibited in xenografts with BAP31-depleted HeLa cells compared with the scramble controls (Fig. 5d, e). Taken together, these findings indicated that BAP31 is essential factor for cervical cancer progression and metastasis.

### BAP31 affects cells migration and invasion by regulating the expression and subcellular localization of the metastasis-related proteins Drebrin, M-RIP, SPECC1L, and Nexilin

**Fig. 6:**
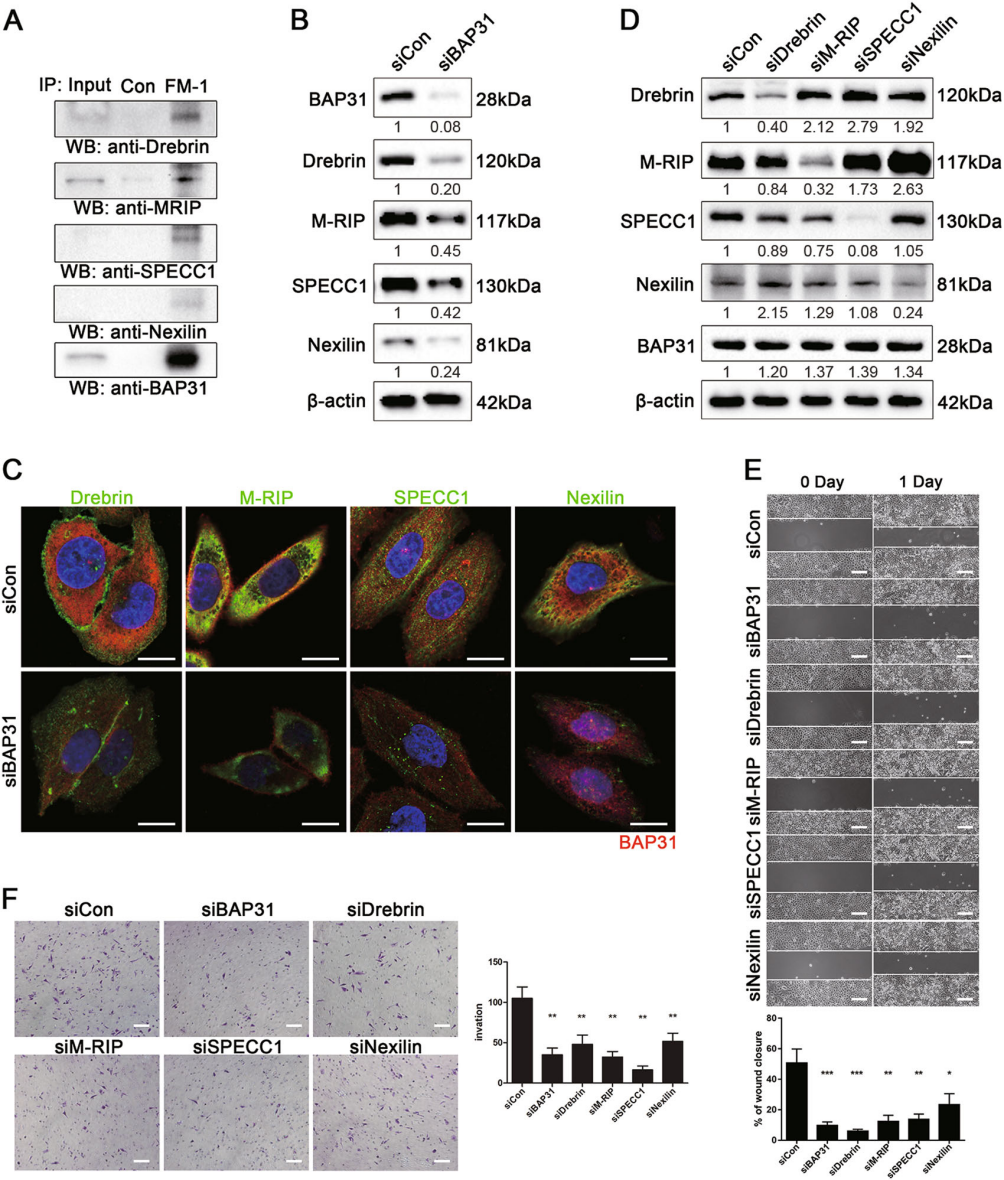
BAP31 affect cells migration and invasiveness by regulating the expressions and subcellular localization of the metastasis-related proteins Drebrin, M-RIP, SPECC1L, and Nexilin. **a** Co-immunoprecipitation experiments showing the interaction between BAP31 and metastasis-related proteins, such as Drebrin, M-RIP, SPECC1L, and Nexilin. HeLa cells extracts were immunoprecipitated with FM-1 and an irrelevant control antibody. The immunoprecipitates were analyzed by western blotting using anti-BAP31, anti-Drebrin, anti-M-RIP, anti-SPECC 1, and anti-Nexilin antibodies. **b** Western blotting results to detect the expression levels of Drebrin, M-RIP, SPECC 1, and Nexilin in control and BAP31-depleted groups of HeLa cells. **c** Immunofluorescence staining of HeLa cells with anti-BAP31, anti-BAP31, anti-Drebrin, anti-M-RIP, anti-SPECC 1, and anti-Nexilin antibodies in control and BAP31-depleted groups. The scale bars represent 5 μm. **d** Western blotting results showing the expression levels of BAP31 in HeLa cells transfected with Drebrin, M-RIP, SPECC1L, or Nexilin siRNA. **e** Wound-healing assays were performed to evaluate the migration of HeLa cells transfected with BAP31, Drebrin, M-RIP, SPECC1L, and Nexilin or control siRNA. The scale bars represent 100 μm. **f** Transwell invasion assays of HeLa and SiHa cells treated as in (**e**). The results represent means ± SD from five microscopic fields. The scale bars represent 100 μm. **p < 0.01 was considered significantly different for each group vs siCon group

To determine the mechanisms by which BAP31 affects cancer metastasis, we performed co-immunoprecipitation assay with FM-1 and analyzed the potential interacting protein by mass spectrometry. We noticed that several proteins regulating actin cytoskeleton organization (Drebrin, M-RIP, SPECC1L, and Nexilin) were enriched in the mass spectrometry data. Interestingly, F-actin-binding protein (Drebrin, SPECC1L, and Nexilin) and upstream cytoskeletal modulator (M-RIP) play critical role in actin cytoskeletal remodeling and are involved in cancer metastasis^22–24^. Considering that actin cytoskeleton dynamics are critical components of the cell migration and invasion process, we surmised that BAP31 might effects the degradation or localization of those metastasis-related proteins. In order to confirm the interaction between BAP31 and Drebrin, M-RIP, SPECC1L, and Nexilin, HeLa cells were extracted and co-immunoprecipitated with FM-1 and the precipitates were analyzed by western blotting with the candidate molecular antibody. We observed that BAP31 co-immunoprecipitated with Drebrin, M-RIP, SPECC1L, and Nexilin (Fig. 6a).

**Fig. 7:**
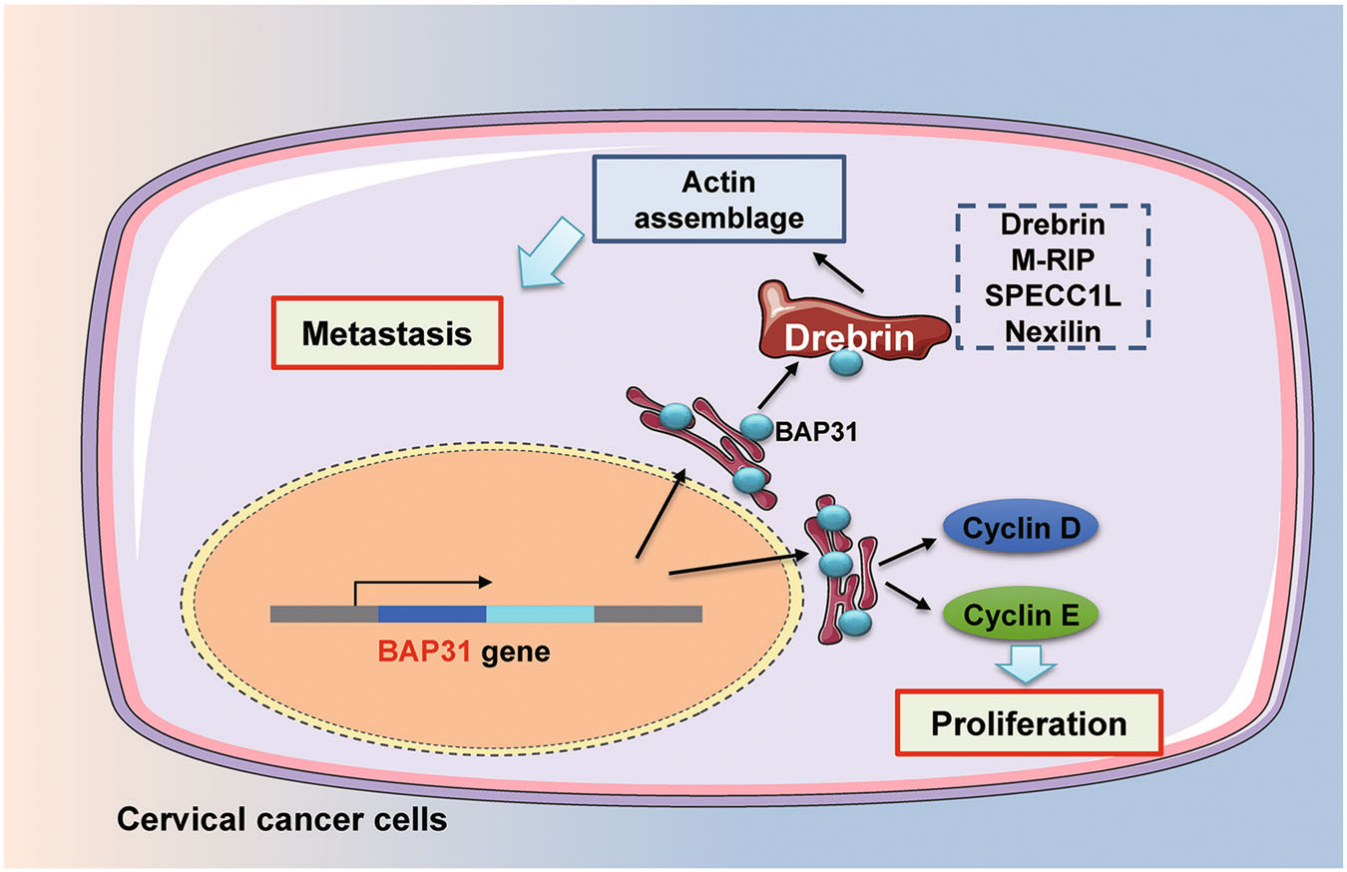
Proposed model of the role of BAP31 in the pathogenesis of cervical cancer. Upregulated BAP31 in cervical cancer first strengthen the cell proliferation by upregulating the cell cycle protein. In addition, BAP31 could affect the assemblage cytoskeleton and promote the metastasis by regulating the expression and subcellular localization of the metastasis-related proteins Drebrin, M-RIP, SPECC1L, and Nexilin

To further examine the correlation between BAP31 and the four metastasis-related proteins, we transiently knocked down BAP31 expression in HeLa cells with siRNA and then analyzed Drebrin, M-RIP, SPECC1L, and Nexilin expression by western blotting and immunofluorescence (Fig. 6b, c). Depletion of BAP31 expression in HeLa cells resulted in the decreased expression of Drebrin, M-RIP, SPECC1L, and Nexilin with a uniform distribution in the cells, indicating that BAP31 could regulates the expression and subcellular localization of these four metastasis-related proteins. Next, we examined whether BAP31 affects the migration and invasion of HeLa cells by regulating the expression of Drebrin, M-RIP, SPECC1L, and Nexilin. HeLa cells were transfected with specific siRNAs-targeting Drebrin, M-RIP, SPECC1L, or Nexilin. Western blotting demonstrated that knockdown of Drebrin, M-RIP, SPECC1L, and Nexilin in HeLa cells had no effect on the expression of BAP31 (Fig. 6d). However, the migration and invasiveness of HeLa cells after Drebrin, M-RIP, SPECC1L, or Nexilin siRNA transfection were decreased compared with control siRNA, which were similar to those depleted of BAP31 (Fig. 6e, f). Taken together, these results indicated that BAP31 affects the migration and invasiveness of cervical cancer cells by regulating the expression and subcellular localization of the metastasis-related proteins Drebrin, M-RIP, SPECC1L, and Nexilin (Fig. 7).

## Discussion

In this study, we developed a novel method to search for novel CTAs, the SADA method. The present study is the first to demonstrate that BAP31 is a novel CTA that is correlated with the progression of clinical cervical cancer and tumor metastasis. Furthermore, BAP31 affects cervical cancer processes mainly by regulating cell cycle-related proteins and cancer cell proliferation. In addition, BAP31 can regulate the metastasis of cervical tumors, preferably by modulating the expression and subcellular localization of the metastasis-related proteins Drebrin, M-RIP, SPECC1L, and Nexilin. These results suggest that BAP31, as a novel CTA, is an effective marker for cancer diagnosis and for prognostic prediction in cervical cancer.

CTAs are immunogenic, highly cancer-specific, and frequently expressed in various types of cancers^1^. Thus, CTAs are promising candidate targets for cancer immunotherapy, including cancer vaccination, adoptive T-cell transfer with chimeric T-cell receptors and antibody-based onco-CTA immunotherapy. Many approaches have been developed to identify unknown CTAs, including T-cell epitope cloning^2^, serological analysis of complementary DNA (cDNA) expression libraries^25^, representational difference analysis^26^, cDNA microarray analysis and massively parallel signature sequencing^27^. However, both immunologically based methods and gene difference analysis have limitations that restrict the efficiency of identifying new CTAs. Normally, cancer patients are in a state of immune suppression, which hinders the identification of CTAs using immunologically based methods. Gene difference analyses are restricted to the differences in the mRNA or protein expression level of specific proteins. Here, we developed SADA as an innovative new method to identify unknown CTAs. Antibodies against spermatogenic cells were first generated, and then candidate antibodies against CTAs were identified by immunohistochemical staining on tumor tissue arrays. Five candidate antibodies with CTA expression patterns were identified. Considering that FM-1 had the highest positive rate than other four antibodies in several types of tumor tissues, especially in cervical cancer tissue, we mainly focused on investigated the target of FM-1 and its potential function in cancer.

BAP31 is highly expressed in primary hepatocellular carcinoma^28^. Our findings demonstrate that BAP31 can function as a useful biomarker for the diagnosis and prognosis of several types of cancers. BAP31 protein level are aberrantly high in cervical, ovarian, breast, liver, esophageal, rectal, and lung cancers. Strikingly, both analysis of our clinical samples and Oncomine data sets demonstrated that BAP31 is associated with poor prognosis in cervical cancer, breast cancer, gastric cancer, and lung cancer. Furthermore, BAP31 expression levels were correlated with cervical cancer metastasis. These results suggest that BAP31 serves as a CTA and might be involved in the progression of several types of cancer.

BAP31 is a chaperone that locates in the ER. The main function of BAP31 is to export secreted membrane proteins from the ER, refer abnormally folded proteins to the ER-associated degradation pathway and regulate cell apoptosis via a caspase-dependent pathway^29,30^. BAP31 is required to maintain HPV-positive cells in a proliferation-competent state by binding to HPV E5^21^. We find that BAP31 is significantly induced in cervical cancer cells and is responsible for the expression of cell cycle-related proteins that then affect the proliferation of cervical cancer cells. These results are consistent with human embryonic stem cells (hESCs) development research indicating that BAP31 affects (hESCs) adhesion, stemness, and survival by regulating proliferation and apoptosis^20^. Moreover, depletion of BAP31 in HeLa cells significantly inhibits the tumor development in mice, which indicates a pro-tumor role in cervical cancer.

In addition to the regulation of proliferation, BAP31 plays an important role in tumor metastasis. Both analysis of our clinical samples and Oncomine data demonstrated that abnormally high level of BAP31 expression are associated with distant metastasis. Studies in xenograft mouse models confirmed the inhibition of tumor metastasis after depletion of BAP31 in cervical cancer cells, suggesting that BAP31 may regulate tumor metastasis. Because the BAP31 protein mainly shuttles between the ER and Golgi, we determined that BAP31 could regulate the expression and subcellular localization of Drebrin, M-RIP, SPECC1L, and Nexilin, which play critical role in actin cytoskeletal remodeling and are involved in cancer metastasis^22–24^. These results indicate that BAP31 inhibits tumor metastasis by selectively binding and regulating the expression of multiple metastasis-related proteins.

Abnormal CTA expression has been recognized as an important event in cancer, and several CTAs have been used as therapeutic targets in antitumor studies. Our data demonstrate that novel CTA BAP31 can inhibit tumorigenesis and progression by regulating multiple oncogenic pathways. Furthermore, recent work in our laboratory indicates that the DNA-vaccine target BAP31 can induce effective immunity against BAP31-expressing malignant melanoma^31^. Taken together, these data indicate a potential role of BAP31 in future anticancer therapy. However, it is not clear why upregulated of BAP31 in cervical cancer and other carcinoma tissues was observed in this study. The mechanism of BAP31 upregulation in cancer remains to be fully elucidated.

In summary, we developed a novel method for identifying unknown CTAs and successfully identified five new CTAs. This method may be useful for identifying highly immunogenic and cancer-specific CTAs for use in diagnosis and immunotherapy. Moreover, we confirmed that BAP31 is a novel CTA and is essential for cervical cancer cells to maintain or promote their abnormal proliferation and metastasis (Fig. 7). These results highlight the potential role for BAP31 as a potential diagnostic and prognostic target for cervical cancer. In addition, inhibition of the expression of BAP31 may provide a new target for the treatment of cancer, but these possibilities require further study. Taken together, these findings provide a new method for detecting unknown CTAs as well as mechanistic insights into how BAP31 regulates cervical cancer hyper-proliferation and metastasis.

## Materials and methods

### Clinical tissue sample collection

Testicular tissues were obtained from a prostate cancer patient after cardiac death. Primary cervical carcinomas and adjacent normal cervical tissue were obtained from 161 patients with clinically and pathologically confirmed cervical carcinomas at Xijing Hospital, the Fourth Military and Medical University (Xi’an, China). Parts of tissue samples were fixed in formalin, embedded in paraffin and sectioned at 4 μm. Other parts of tissues were stored in liquid nitrogen and prepared for protein or mRNA extraction. 107 of the cervical cancer patients received the telephone follow-up for the overall survival analysis, and 54 patients were loss to follow-up. All samples were collected with informed consent from all subjects. This study was approved by the local medical research ethics committee of The Fourth Military Medical University, Xi’an, China.

### Spermatogenic cells separation, mice immunization, and generation of anti-testis monoclonal antibodies

In brief, donated testicular biopsies were sliced into small pieces and digested with two enzymes: 1 g/L type I collagenase (Sigma-aldrich) for 20 min and trypsin-ethylenediaminetetraacetic acid (EDTA) (0.25%) for 15 min. The disassociated cell were filtered through 200-mesh filtration traps and then collected by centrifugation. Percoll density gradient centrifugation was performed to purify the human spermatocytes/spermatogonia. Percoll was diluted to gradient concentrations of 11, 19, 27, 35, and 43% with phosphate-buffered saline (PBS) and added to the bottom of the centrifuge tube from the highest concentration to the lowest concentration. The newly collected cell suspension was added slowly to the top layer of the Percoll separation medium followed by centrifugation at 600 × *g* for 30 min. Cells present at a gradient concentration ranging between 30 and 40% Percoll were collected as the spermatocytes/spermatogonia. The cell purity was measured by immunocytochemical staining with LAGE-1 antibody.

Spermatocytes/spermatogonia (1 × 10^6^/mL) were injected intraperitoneally into BALB/c mice. The same spermatocytes/spermatogonia were injected intraperitoneally twice after 4 weeks and 7 weeks. Immunohistochemical staining of testis sections were performed to determine the serum titers of the immunized mice. Ten days after cell immunization, splenocytes from the boosted mice and SP20 myeloma cells cultured in Rosewell Park Memorial Institue (RPMI) 1640 medium containing 20% fetal calf serum (FCS) were fused in the presence of PEG (MW4000, Merck, Darmstadt, Germany) and cultured in RPMI 1640 medium containing 20% FCS and HAT (Invitrogen, Carlsbad, CA). The positive hybrids were selected and then subcloned three times using a limiting dilution method.

BALB/c mice were injected with 0.2 mL of pristane and 7–10 days later, the mice were inoculated with hybridoma cells (1 × 10^6^/mL). The ascitic fluid was collected after 7–14 days. The immunoglobulins fraction was precipitated from the ascetic fluid using 50% ammonium sulfate and then subjected to affinity purification using Protein G-Sepharose CL-4B (GE Healthcare, Piscataway, NJ) chromatography. The IgG fractions were pooled together and dialyzed against PBS. A aliquots of the purified antibodies were stored at − 70 °C.

### Cell culture, transfection, and xenograft

The human cervical carcinoma cell lines HeLa and SiHa (ATCC) were cultured at 37 °C with 5% CO_2_ in Dulbecco’s Modified Eagle’s Medium (Gibco) and Minimum Essential Media (Gibco), respectively, supplemented with 10% FBS (Hyclone). CHO cells were obtained from the Cell Bank of the Chinese Academy of Sciences and grown using standard protocols. To generate the purified BAP31 protein, the open reading frame was cloned into the CMV3 plasmid with GST, and the resulting vector was transfected into CHO cells.

RNAi studies were performed by using siRNAs-targeting BAP31, Drebrin, M-RIP, SPECC1L, or Nexilin specifically or control siRNA with Lipofectamine 3000 reagent according to the manufacturer’s instructions. Specific siRNA sequences are given in Supplementary Table S2. Stable BAP31-depletion HeLa cells were prepared as previously reported. BAP31 shRNA, including BAP31 siRNA and a reverse complement of the same sequence linked with a nine-nucleotide non-complementary spacer (tctcttgaa), were cloned into pSUPER.retro.circular.stuffer vector and the result vector were transfected into HeLa cells. Puromycin was used as a selection maker for the creation of stable BAP31-depleted HeLa cells and BAP31 expression was confirmed by real-time PCR and western blotting.

For tumor xenograft, stable BAP31-depletion HeLa cells were injected subcutaneously (1 × 10^6^ cell per site) into the right flanks of nude mice (6–8 weeks old) (Department of Laboratory Animal Medicine of the Fourth Military Medical University). HeLa cells transfected with control shRNA were used as controls and five mice were included in each group. Mice was examined three times a week for development. The size of tumors was measured using calipers and calculated as fellow: (large diameter) × (short diameter)^2^/2 and expressed in mm^3^. This study was approved by the institutional review board, the fourth military medical university.

### Quantitative real-time PCR analysis

Total RNA from cells or tissues was extracted by using Trizol (Takara) according to the manufacturer’s instruction. cDNAs were then generated using a PrimeScript RT regent kit with 1 μg of total RNA per reaction. Quantitative real-time PCR was conducted using the SYBR Premix Ex Taq. *GAPDH* was used as an internal control. Samples were normalized to the independent control housekeeping gene β-actin and were reported according to the ΔΔCT method as RNA fold increase: 2^ΔΔCT^ =2^ΔCT sample^−2 ^ΔCT reference^. The sequences of each primers are listed in Supplementary Table S2.

### Western blot analysis

Cells or biopsies were homogenized in radioimmunoprecipitation assay reagent with phenylmethylsulfonyl fluoride (protease inhibitors mix) (Sigma-Aldrich, USA). Proteins (20–50 μg) were separated by sodium dodecyl sulfate polyacrylamide gel electrophoresis and transferred to PVDF membranes. After blocking for 2 h with blocking buffer (1 × Tris-buffered saline with 5% skim milk and 0.01% Tween-20), the membranes were treated with the primary antibodies, including anti-BAP31, anti-Drebrin, anti-M-RIP, anti-SPECC1L, anti-Nexilin, anti-cyclin D, anti-cyclin E1, and anti-cyclin E2 (Abcam, Cambridge, MA), overnight at 4 °C. Membranes were washed with tris-buffered saline and then incubated with horseradish peroxidase (HRP)-conjugated secondary antibodies for 1 h at room temperature. Proteins were detected using a chemiluminescence detection kit (KPL, Gaithersburg, MD). The signal intensities of western blots were measured quantitatively using the Image J software and β-actin was used as a loading control.

### Immunohistochemistry staining

In brief, ~ 4-μm paraffin sections were stained with hematoxylin–eosin, mouse anti-FM-1, or rabbit anti-BAP31 antibodies. Sections were blocked with 3% H_2_O_2_ to eliminate endogenous tissue peroxidase followed by heat-mediated antigen retrieval with Tris/EDTA (pH 9.0) buffer. Specimens were incubated with anti-FM-1 or anti-BAP31 antibodies overnight at 4 °C. HRP-labeled secondary antibodies were applied to the sections for 1 h at room temperature before immunostaining with DAB chromogen (Dako). Isotype antibodies were used as the negative control. The HE and immunohistochemical staining were digitalized with the Nanozoomer Digital Pathology System (Hamamatsu, Herrsching, Germany). Specimens were reviewed for staining intensity and staining extent.

The evaluation of staining scores was described previously [21]. In brief, the percentages of staining-positive cells were scored into four categories: 0 (0%), 1 (1–33%), 2 (34–66%), and 3 (67–100%). The staining intensities were scored into four grades: 0 (none), 1 (week), 2 (moderate), and 3 (strong). The final staining score was defined as the product of the percentage and intensity scores.

### Immunofluorescence confocal microscopy

HeLa cells were grown on coverslips in six-well plates and then transfected with siRNA as described above. The coverslips were rinsed twice with 0.01 mol/L PBS and fixed with acetone at 4 °C for 10 min. The cells were next incubated in 0.3% Triton X-100 for 5 min at room temperature. Non-specific interactions were blocked with 4% bovine serum albumin at 37 °C for 30 min. After rinsing three times with 0.01 mol/L PBS, the cells were incubated with anti-BAP31, anti-Drebrn, anti-M-RIP, anti-SPECC1L, or anti-Nexilin mAb overnight at 4 °C, followed by a secondary CY3-labeled secondary antibody for 1 h at 37 °C in the dark. 4′,6-diamidino-2-phenylindole was applied to all cells for nuclear counterstaining. Samples were analyzed by confocal microscopy using an FV-1000/ES confocal microscope (Olympus, Tokyo, Japan).

### Colony-formation assay

The cancer cells that were transfected with BAP31 siRNA or control siRNA (1000 cells per well) were seeded into six-well plates and cultured in a humidified incubator for 10 days. Colonies were fixed with methanol and stained with crystal violet for 15 min at room temperature. Cancer cell proliferation was evaluated in five microscopic fields. All experiments were repeated at least three times.

### Scratch wound-healing assay

The cancer cells that were transfected with BAP31-specific siRNA or control siRNA were cultured into six-well cell plates. When the cell confluence reached 90%, 1 µg/ml mitomycin C was added for 1 h to inhibit cells proliferation and then a straight scratch was made with pipette tips. Images were continuously captured at the same site of the scratch at 0, 12, and 24 h after the wounding. All experiments were repeated at least three times.

### Cell invasion assay

Filters (8 μm pore size) pre-coated with fibronectin and Matrigell (BD) in 24-well chambers were used to estimate cancer cell migration. HeLa and SiHa cells transfected with specific siRNAs were detached, and the cell suspension was placed into the upper chamber in 600 μL of serum-free Dulbecco's Modified Eagleʼs Medium (DMEM) (1 × 10^5^ cells per filter). DMEM medium supplemented with 10% FBS was placed in the lower chamber as a chemoattractant. HeLa and SiHa cells were cultured for 26 and 18 h, respectively. The cells at the lower surface of the filter were fixed in methanol and stained with crystal violet. Cancer cells invasion was expressed as the mean number of five microscopic fields. All experiments were repeated at least three times.

### Bioinformatics analysis

The RNA-Seq data of patients in TCGA-LIHC project were downloaded from Genomic Data Commons Data Portal, as well as the corresponding clinical information. Differential expression analysis, GO, KEGG, Disease Ontology enrichment analysis were performed with R language (version3.3.2). The GSEA was performed on the expression data of 303 cervical cancer patients. Nominal *p* value was used to estimate the statistical significance of the enrichment score.

### Statistical analysis

All the experiments were repeated at least three times, and statistical analyses were performed using GraphPad Prism 5.0 (GraphPad Software, San Diego, CA). For experiments with more than two groups, the differences between groups were compared by a one-way analysis of variance followed by Dunnett’s test, in which all groups were tested against a control group as a reference. For experiments with only two groups, Student’s *t* test was used for comparisons of group means. Survival was analyzed by the Kaplan–Meier and log-rank *t* test. *p* values < 0.05 were considered to represent significant differences.

## Electronic supplementary material

SUPPLEMENTAL MATERIAL
