# Supplementary material for: BAP31, a newly defined cancer/testis antigen, regulates proliferation, migration, and invasion to promote cervical cancer progression
Source: Cell Death Dis. 2018 Jul 18;9(8):791. doi: 10.1038/s41419-018-0824-2 (PMC6052025; doi:10.1038/s41419-018-0824-2)
Supplement: Supplementary file 1 — SUPPLEMENTAL MATERIAL [file 41419_2018_824_MOESM1_ESM.docx]

**BAP31, a newly defined cancer/testis antigen, regulates proliferation, migration and invasion to promote cervical cancer progression**

Erle Dang^1,3,*^, Shuya Yang^1,*^, Chaojun Song^1,4,*^, Dongbo Jiang ^1,*^, Zichao Li, Wei Fan^2^, Yuanjie Sun^1^, Liang Tao^1^, Jing Wang ^1^, Tingting Liu ^1^, Chunmei Zhang^1^, Boquan Jin^1^, Jian Wang^2^, Kun Yang^1^

^1^Department of Immunology, the Fourth Military Medical University, Xi’an 710032, Shaanxi, People’s Republic of China

^2^Department of Obstetrics and Gynecology, Xijing Hospital, the Fourth Military Medical University, Xi'an 710032, Shaanxi, People’s Republic of China.

^3^Department of Dermatology, Xijing Hospital, the Fourth Military Medical University, Xi’an 710032, Shaanxi, People’s Republic of China

^4^School of Life Science, Northwestern Polytechnic University, Xi’an, 710072, Shaanxi, People’s Republic of China.

^*^These authors contributed equally to this work.

Corresponding author: E-mail: [yangkunkun@fmmu.edu.cn](mailto:yangkunkun@fmmu.edu.cn) (Kun yang), [wangjian_fmmu@163.com](mailto:wangjian_fmmu@163.com) (Jian Wang) or immu_jin@fmmu.edu.cn (Boquan Jin). Phone: +86-029-84779171. Fax: +86-029-84779171

**
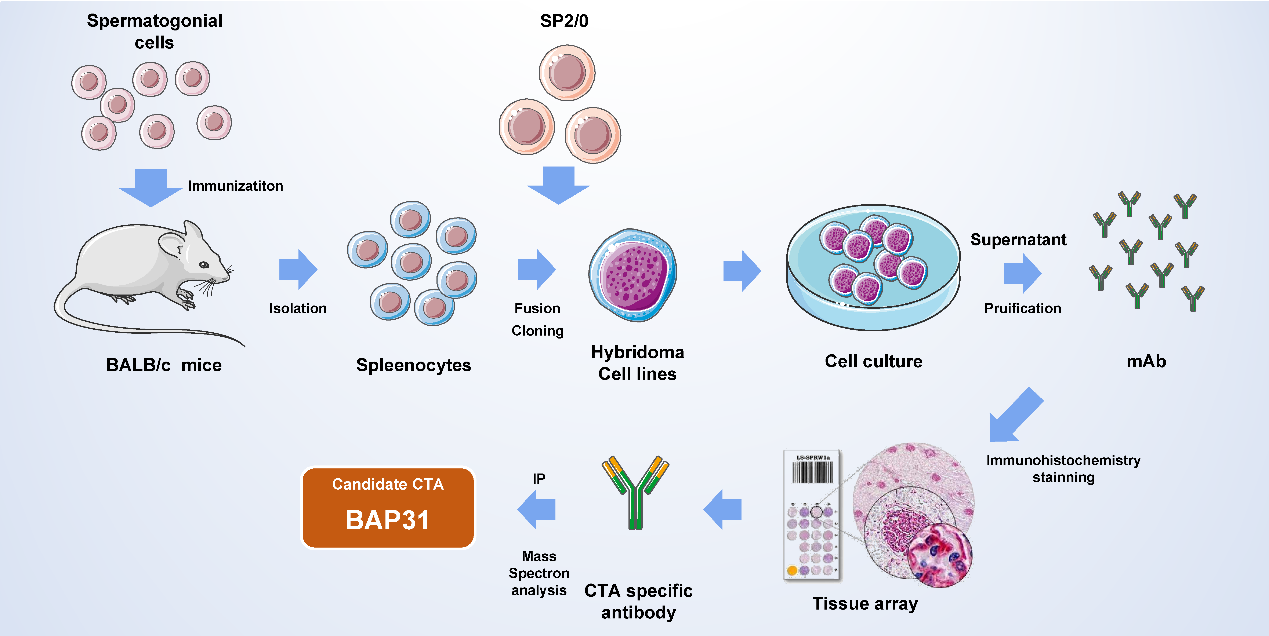
**

**Supplementary Figure 1. Flowchart of the spermatogenic-cells-specific monoclonal-antibody-defined cancer/testis antigen (SADA) method**

**
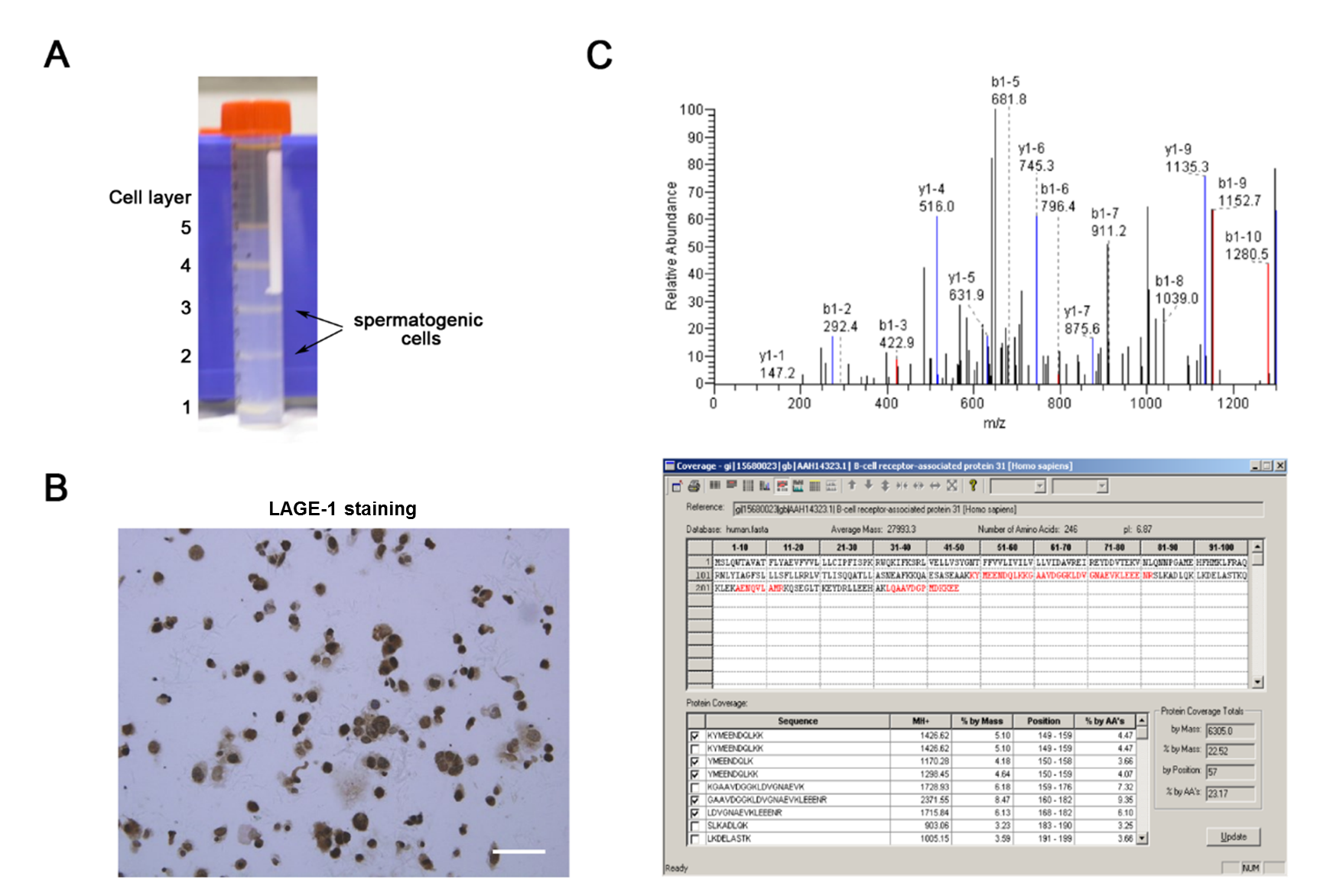
**

**Supplementary Figure 2. Identification of BCAP31 as a new CTA using SADA method.**

(A) Purification of human spermatocytes/spermatogonia from donated testis tissue using Percoll density gradient centrifugation. (B) Immunocytochemical staining shown the spermatocytes-spermatogonia cell purity with LAGE-1 antibody. (C) Mass spectrometry analysis identified that BCAP31 was the molecular recognized by FM-1. The scale bars represent 100 μm.


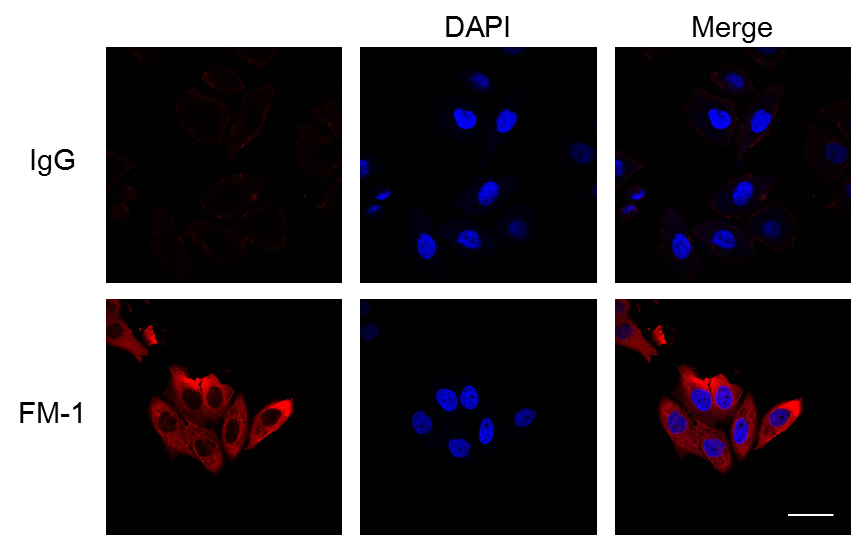


**Supplementary Figure 3. HeLa cell was positive stained using immunofluorescence staining with FM-1.**

Immunofluorescence staining of HeLa cells with FM-1. IgG were used as a negative control. The scale bars represent 10 μm.


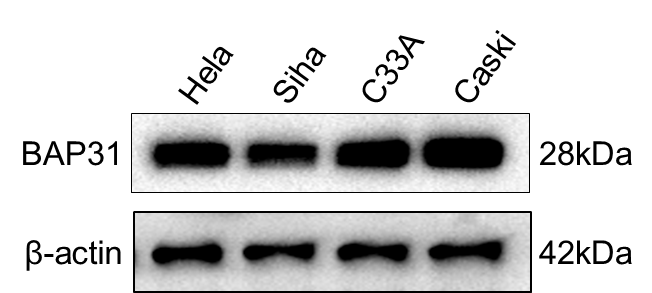


**Supplementary Figure 4. BAP31 expression in cervical cancer cell lines Hela, Siha, C33A and Caski.**

Western blotting results to detect the expression levels of BAP31 in Hela, Siha, C33A and Caski.

**Supplementary Table S1. Characteristic of 5 candidate CTA antibodies**

| Code | Subclass | Expression pattern | Localization | IHC results on tumor sections | FCM |
| --- | --- | --- | --- | --- | --- |
| FM-1 | IgG1 | Early stage | Cytoplasm | Carcinoma of esophagus, colon, rectum, thyroid, lung, prostate, breast, cervix, ovary, skin, pancreas | + |
| FM-2 | IgG1 | Middle stage | Cytoplasm | - | - |
| FM-3 | IgG2a | Full stage | Membrane | - | - |
| FM-4 | IgG1 | Full stage | Membrane | - | - |
| FM-5 | IgG1 | Full stage | Membrane | Carcinoma of esophagus, lung, urinary bladder, kidney, cervix, ovary | + |
| FM-6 | IgG1 | Early stage | Cytoplasm | Carcinoma of esophagus, liver, colon, rectum, thyroid, lung, urinary bladder, kidney, cervix, ovary | - |
| FM-7 | IgG1 | Middle stage | Cytoplasm | - | - |
| FM-8 | IgG3 | Late stage | Cytoplasm | - | - |
| FM-9 | IgG2b | Middle stage | Cytoplasm | - | - |
| FM-10 | IgM | Early stage | Nucleus | Carcinoma of esophagus, stomach, liver, colon, rectum, thyroid, lung, urinary bladder, kidney, prostate, breast, cervix, ovary | - |
| FM-11 | IgG2b | Full stage | Membrane | - | - |
| FM-12 | IgG1 | Late stage | Cytoplasm | - | - |
| FM-13 | IgG1 | Full stage | Membrane | - | - |
| FM-14 | IgG1 | Full stage | Membrane | - | - |
| FM-15 | IgG1 | Full stage | Membrane | - | - |
| FM-16 | IgG2b | Full stage | Membrane | - | - |
| FM-17 | IgG1 | Middle stage | Membrane | - | - |
| FM-18 | IgG1 | Early stage | Cytoplasm | Carcinoma of esophagus, colon, rectum, thyroid, lung, urinary bladder, cervix, ovary, skin | - |
| FM-19 | IgG1 | Late stage | Cytoplasm | - | - |

**Supplementary Table S2. Primers and Sequences Used in the Experiment**

| **Primers for Real Time PCR** | |
| --- | --- |
| Homo sapiens BCAP31 |  |
| Forward | CGGCTGGTGGAGTTGTTAGT |
| Reverse | CGGGATTGTTCTGGAGGTT |
| GAPDH |  |
| Forward | GACCTGACCTGCCGTCTA |
| Reverse | AGGAGTGGGTGTCGCTGT |
| **SiRNA** |  |
| BCAP31 shRNA |  |
| Sense | gatccccggtgaacctccagaacaatttcaagagaattgttctggaggttcacctttttggaaa |
| Antisense | agcttttccaaaaaggtgaacctccagaacaattctcttgaaattgttctggaggttcaccggg |
| BCAP31 siRNA |  |
| Sense | ggtgaacctccagaacaat |
| Antisense | attgttctggaggttcacc |
| Drebrin siRNA |  |
| Sense | GACACCAUUGAAACUGACA |
| Antisense | CUGUGGUAACUUUGACUGU |
| M-RIP siRNA |  |
| Sense | CCGACCUGCUGAAUUUCAA |
| Antisense | GGCUGGACGACUUAAAGUU |
| SPECC1 siRNA |  |
| Sense | GGCACAGCUUCUUCAACCA |
| Antisense | CCGUGUCGAAGAAGUUGGU |
| Nexilin siRNA |  |
| Sense | CAGUCAGAUUUACGGUUAA |
| Antisense | GUCAGUCUAAAUGCCAAUU |
| NC siRNA |  |
| Sense | UUCUCCGAACGUGUCACGUTT |
| Antisense | ACGUGACACGUUCGGAGAATT |
